# Research on the Grinding Energy Density in a Jet Mill

**DOI:** 10.3390/ma14082008

**Published:** 2021-04-16

**Authors:** Dariusz Urbaniak, Henryk Otwinowski, Tomasz Wyleciał, Vladimir Pavlovich Zhukov, Aleksei Yevgenyevich Barochkin, Jarosław Boryca

**Affiliations:** 1 Department of Thermal Machinery, Faculty of Mechanical Engineering and Computer Science, Czestochowa University of Technology, 42-201 Czestochowa, Poland; urbaniak@imc.pcz.pl; 2 Department of Production Management, Faculty of Production Engineering and Materials Technology, Czestochowa University of Technology, 42-201 Czestochowa, Poland; tomasz.wylecial@pcz.pl (T.W.); jaroslaw.boryca@pcz.pl (J.B.); 3 Department of Applied Mathematics, Faculty of Electromechanics, Ivanovo State Power Engineering University, 153003 Ivanovo, Russia; zhukov-home@yandex.ru; 4 Department of Electric Power Systems Automatic Control, Faculty of Electrical Power Engineering, Ivanovo State Power University, 153003 Ivanovo, Russia; admin@tes.ispu.ru

**Keywords:** comminution, grinding energy, jet mill

## Abstract

Raw materials are used in many industrial technologies. The raw material frequently has to be prepared as an intermediate with an appropriate particle size distribution, which requires the use of grinding. In grinding processes, energy consumption is a very important profitability criterion for the applied particular size reduction technology. The paper describes the comminution process that takes place in the jet mill using a modified form of the thermodynamic theory of grinding. In this theory, new material characteristics have been added: the surface and volumetric density of grinding energy. The thermodynamic theory is a combination of the classical Kick’s theory and the modified form of Rittinger’s theory. The tested physical magnitudes are a measure of the energy consumption of the grinding process. They describe the energy that must be provided in the grinding process to overcome interactions between particles related to the volume and surface of the material. Knowledge of these magnitudes is necessary to model thermomechanical phenomena in the solid state. The paper presents the results of research on comminution in a jet mill, on the basis of which the values of the tested material magnitudes were determined. It is graphically shown how the values of the tested magnitudes depend on the grain size of the ground samples.

## 1. Introduction

Currently, there are efforts to implement technological processes that would fit into the model of the closed cycle economy. The existing models of manufacturing of various goods have not always led one to seek savings, both in terms of the used raw materials and energy saving. The product of such manufacturing processes was valuable as long as it met specific needs. After this period, it became a waste and most often was subjected to the disposal process without also analyzing the problem of the recovery of its possible material components and energy. Such an approach has a significant influence on the economy. The faster the economy started to gallop in its development, the faster it led to the depletion of raw materials and a growing demand for energy.

It was then necessary to stop these trends, resulting in the development of assumptions for the circular economy [1,2,3]. The idea of a new way of looking at the process of manufacturing goods has become, among other things, the tendencies to look for raw materials and energy savings. The process of manufacturing new goods began to include as many components of already used goods as possible in order to save primary raw materials. For example, [4] presents the possibility of using lime waste in the production of alternative sorbents for flue gas desulphurization. Unnecessary goods have come to be seen in the context of the potential recovery of energy used in the prior generation phase in order to save the amount of energy needed to produce a new good. Another aspect of the circular economy is looking for solutions that reduce the energy consumption of production processes for their energy optimization.

In industrial technologies, raw materials are used that must meet a number of requirements: a homogeneous chemical composition, specific properties obtained by heat treatment, a defined geometric shape that is the effect of machining, etc. The raw material frequently has to be prepared as an intermediate with an appropriate granularity [5], which requires the use of grinding. Biobased materials such as wood or plant particles will have a significant length-to-width ratio because during grinding the material will break more easily perpendicular to the fiber than along the fiber direction. This is due to the orthotropic viscoelastic properties of biobased materials [6]. The influence of lignocellulosic biomass milling on the properties of particles (size, shape, surface area) is discussed in [7].

In grinding processes, energy consumption is a very important profitability criterion for the applied particular size reduction technology. The energy consumption of grinding is the subject of many studies, especially in the case of ball mills for grinding clinkers [8,9], fuels [10], iron ore [11], hematite ore [12], sulfide ore [13], wet ores [14], and in the case of stirred mills for grinding ores [15,16,17] and wet quartz [18]. In [19,20], the energy efficiency of various coals grinding in the ball-and-race mills is studied experimentally. The grinding process of cement in a vertical roller mill over ball mills is optimized by the authors of [21] in order to minimize power consumption. The energy consumption is also studied in semi-autogenous mills [22,23,24], autogenous mills [25] and rotary mills [26]. Biomass grinding is investigated by many researchers. The energy consumption under vegetal biomass grinding is studied using hammer mills [27,28,29,30], multi-disc mills [31], vibratory mills [32] and jet mills [33]. The authors [34] performed the optimization of corn stover grinding in a Wiley knife mill with the use of a hybrid genetic algorithm. Research on energy consumption in the grinding process is also carried out in special mills, for example in an electromagnetic mill [35].

From a theoretical aspect, the relationship that describes the relationship between the energy necessary to carry out the grinding process and the process effect, most often measured by the final surface of the grinding product, is called the grinding theory. The grinding theories, also called hypotheses or laws, are known by the surnames of their authors, such as: Rittinger [36], Kick [37,38,39], Bond [40,41,42] and Charles [43,44,45]. The theory of Rittinger assumes that the energy introduced in the process of grinding once is proportional to the increase in the surface resulting from the formation of new grains. According to Kick, this energy is proportional to the volume of the fed substance. Bond theory combines the hypotheses of Rittinger and Kick, and hence it is often referred to as the surface-volume theory. Charles proposed a generalization of the three theories into one. An alternative formulation of the energy hypotheses was proposed by Djingheuzian [46,47,48]. Djingheuzian’s hypothesis, known as the thermodynamical theory of comminution, was developed by Guillot [49] and Mielczarek [50,51]. Theoretical and experimental analysis performed on the basis of data adopted from the literature shows that Rittinger’s, Kick’s and Bond’s hypotheses can be used for different materials [52]. The grinding theories can also be applied to evaluate the efficiency of the grinding of food materials with varied levels of moisture content [53]. The most popular grinding theories—Kick’s theory and Rittinger’s theory—have a limited range of application. Kick’s theory is used to describe the crushing processes, while Rittinger’s theory is used to describe grinding in mechanical mills [33,52,54,55,56]. The paper describes the comminution process taking place in the jet mill when using a modified form of the thermodynamic theory of grinding, which is a combination of the Kick’s theory and the Rittinger’s theory.

An analysis of the literature shows that continuous research on grinding theories is still needed. These studies are of particular importance for the implementation of technological processes under the aspect of the circular economy. Understanding the complex grinding processes will optimize these processes, saving both raw materials and energy.

## 2. A Modified Form of the Thermodynamic Theory of Grinding

According to the thermodynamic theory of grinding, the energy supplied in the grinding process is proportional to the total volume of the fed material and the surface of the product [50,51].

(1) $$E_{ks1}=\frac{\sigma_{m}^{2}}{2E}V+\alpha \left(A_{1}+\Delta A\right)$$

where: *E_ks_*_1_—initial kinetic energy of the grinding material, J; *σ_m_*—the compressive stress on the verge of destruction, N/m^2^; *E*—Young’s modulus, N/m^2^; *V*—the volume of fed material, m^3^; *α*—the surface density of the grinding energy, J/m^2^; *A*_1_—the surface of the feed, m^2^; Δ*A*—the increase of the total surface of the grinding material, m^2^.

The theoretical value of the breakage strength is determined by crystal structures of brittle solid material. Regular crystals are difficult to observe in the internal structure of solid materials. Microscopic observations show the presence of aggregates with an incorrectly distorted spatial form and randomly distributed clusters of crystals, called crystallites [57,58]. In addition, defects in the internal crystal lattice structure, along with micropores, microcracks, the inclusions of other defects or other minerals, often constitute the natural structure of these materials. The theoretical value of the stress causing the destruction of solid particles is 10^2^–10^4^ times greater than the actual strength [58].

Griffith [59] postulated that in any solid material there are small fissures that weaken its structure. When the appropriate stresses are applied, the fissure increases and the material cracks. According to the model of the weakest link, the material is torn apart if the local breaking stress exceeds the critical one.

Based on the analysis above, a modification of the thermodynamic theory of grinding was proposed:

(2) $$E_{ks1}=\left(\frac{\sigma_{m}^{2}}{2E}-C\right)V+\alpha \left(A_{2}\right)$$

where: *C*—volumetric density of the energy of material weakening as a result of the existence of defects in the internal construction of the substance, J/m^3^; *A*_2_—total final surface of the ground material, m^2^.

The volumetric density of the energy *C* determines the impact of the existing defects in the solid internal structure on the elastic limit strain.

In the case of comminution in a jet mill, absolute physical quantities are not used to describe the process, but their streams:

(3) $${\dot{E}}_{ks1}=\left(\frac{\sigma_{m}^{2}}{2E}-C\right)\dot{V}+\alpha \left(\dot{A_{2}}\right)$$

where: ${\dot{E}}_{ks1}$—stream of the initial kinetic energy of the grinding material, J/s; $\dot{V}$—stream of the volume of material, m^3^/s; ${\dot{A}}_{2}$—stream of the total final surface of the ground material, m^2^/s.

It is assumed that:

(4) $$\frac{\sigma_{m}^{2}}{2E}-C=\beta$$

then:

(5) $${\dot{E}}_{ks1}=\beta \dot{V}+\alpha \left(\dot{A_{2}}\right)$$

## 3. Determination of Material Characteristics

In order to determine the input energy necessary for the grinding process, the following strength material characteristics of brittle substances should be designated:

- the surface density of the grinding energy *α*,
- the volumetric density of the grinding energy *β*.

**Figure 1:**
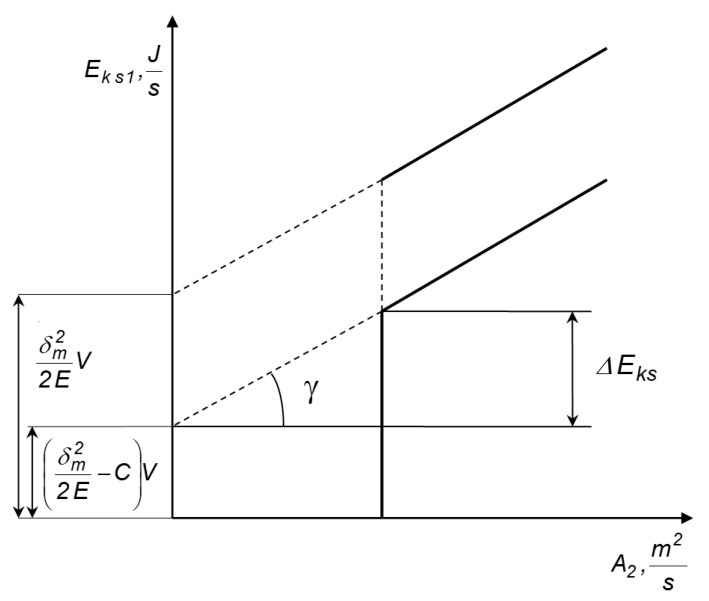
Scheme for determining the elastic limit stress values and the surface density of the grinding energy.

When the volume of the substance is constant, Equations (1) and (3) describe a straight line in the coordinate system ${\dot{E}}_{ks1}-{\dot{A}}_{2}\;$ [50,60]. Then, the material characteristics $\frac{\sigma_{m}^{2}}{2E}\dot{V}$ and $\left(\frac{\sigma_{m}^{2}}{2E}-C\right)\dot{V}$ are the energy values for the surface of the product, equal to *A*_2_ *=* 0 (Figure 1).

From Figure 1, it follows that:

(6) $$\Delta {\dot{E}}_{ks}={\dot{E}}_{ks1}\left({\dot{A}}_{1}\right)-{\dot{E}}_{ks1}\left(0\right)=\alpha {\dot{A}}_{1}+\left(\frac{\sigma_{m}^{2}}{2E}-C\right)\dot{V}-\left(\frac{\sigma_{m}^{2}}{2E}-C\right)\dot{V}=\alpha {\dot{A}}_{1}$$

and:

(7) $$\mathrm{tg}\gamma =\frac{\Delta {\dot{E}}_{ks1}}{{\dot{A}}_{1}}=\frac{\alpha {\dot{A}}_{1}}{{\dot{A}}_{1}}=\alpha$$

Thus, the surface density of the grinding energy, related to the state and physicochemical properties of the ground material, will be equal to the tangent of the angle of inclination of the straight line ${\dot{E}}_{ks1}=f\left({\dot{A}}_{2}\right)$.

## 4. Research in the Jet Mill

**Figure 2:**
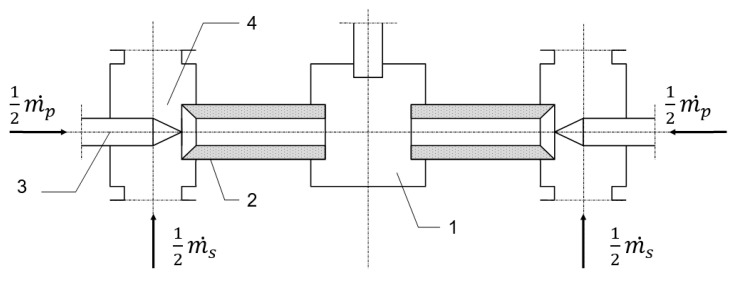
Diagram of the jet mill: (1)—grinding chamber, (2)—acceleration tube, (3)—nozzle and (4)—suction chamber.

This paper presents the results of research on the comminution process in a jet mill. The jet mill presented schematically in Figure 2 allows for impact loading of the material without the cooperation of the elements of the device, which is the result of using the kinetic energy of the colliding grains. The stream of working air ${\dot{m}}_{p}$ is compressed so that its energy is sufficiently high. The compressed medium flows into the nozzles (3) of the grinding chamber (1), where it expands to reach the speed of sound in the nozzles’ outlet section. This allows a negative pressure to develop in the suction chambers (4), which determines the suction of the solid phase material ${\dot{m}}_{s}$. Then, the two-phase gas-solid mixture flows through the acceleration tubes (2), wherein the flow velocity of the gas particle sand solid grains is equalized. The solid grains flowing out of the acceleration tubes have a sufficiently high kinetic energy, which allows, upon a collision with an identical stream flowing from the opposite direction, for the grinding of the material.

The geometric parameters of the jet mill were as follows [61]: nozzle diameter—2.4 mm, diameter of acceleration tube—10 mm, length of acceleration tube—100 mm, diameter of milling chamber—120 mm and distance between acceleration tubes—48 mm. The jet mill consists primarily of metal components that are made of St3S weldable structural steel.

Samples of silica sand with the following grain size ranges: 300–800 μm; 200–500 μm; 100–360 μm; 70–180 μm; and 20–110 μm were used in the study to analyze the modified form of the thermodynamic theory of grinding [62]. The material was subjected to a one-off comminution in the jet mill stand. During the tests, the following parameters were measured: the working air pressure and temperature; the ambient air humidity, pressure and temperature; and the time of the measurement. The values of these parameters were used as the input magnitudes of the basic calculation algorithm. After the tests, the particle size distribution of the feed and comminution products was measured in order to determine the final surface of the products.

## 5. Analysis of the Obtained Results

**Figure 3:**
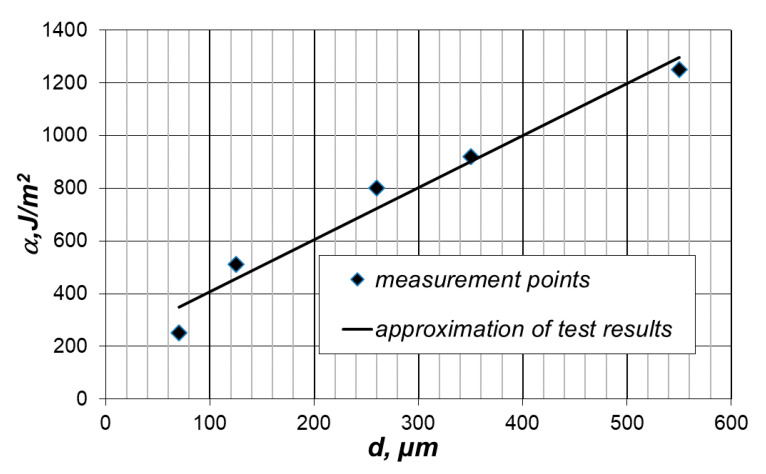
Value of the surface density of the grinding energy as a function of the feed size (average grain size).

**Figure 4:**
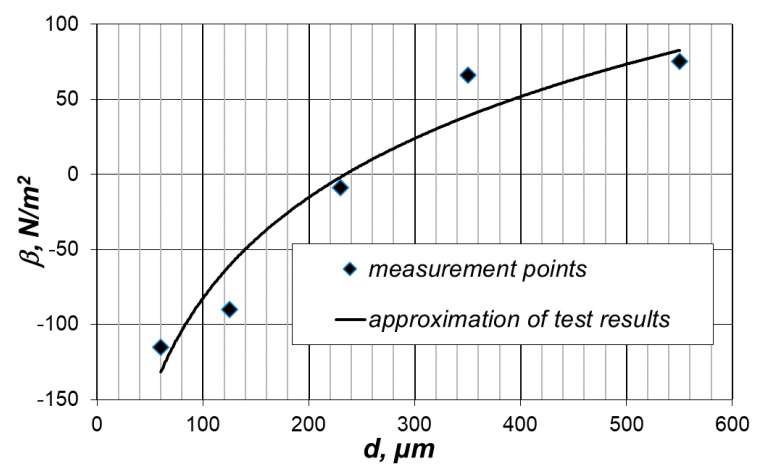
Value of the actual elastic limit stress as a function of the feed size (average grain size).

Using the methodology for determining the material characteristics (Section 3), appropriate calculations were carried out. The values of the material characteristics are shown in Figure 3 and Figure 4.

When analyzing the values of the surface density of the grinding energy for various feed grain sizes, it should be stated that, in this case, the susceptibility to grinding depends on the grain size of the ground material. The values of the surface density of the grinding energy are the highest for the coarsest material. For finer materials, the characteristic value decreases successively with an increase in the value of the average grain size.

The surface density of the grinding energy is described by the relation [50,60,63]:

(8) $$\frac{n_{1}}{a_{V1}}{\bar{\varepsilon }}_{I}=\alpha$$

where: $a_{V1}$—the surface density of the input material, m^2^/m^3^; *n*_1_—the total number of particles on the surface of the ground substance; ${\bar{\varepsilon }}_{I}$—the average value of the bond energy of one surface particle.

(9) $${\bar{\varepsilon }}_{I}=\frac{n_{p}\varepsilon_{p}+n_{k}\varepsilon_{k}+n_{w}\varepsilon_{w}}{n}$$

where: *n =* $n_{p}$ *+* $n_{k}$ *+* $n_{w}$—the total number of particles on the border of phases in a unit of volume; $\varepsilon_{p}$, $\varepsilon_{k}$, $\varepsilon_{w}$—the bond energy of surface particles, edge particles and apex particles, respectively.

Quotient *n*_1_*/*$a_{V1}$ is constant, while its value depends very much on the state of the surface; it fulfills the condition:

(10) $$\left|\varepsilon_{p}\right|\rangle \left|{\bar{\varepsilon }}_{I}\right|\rangle \left|\varepsilon_{w}\right|$$

The averaged energy of bonds of the surface particles  ${\bar{\varepsilon }}_{I}$ will be closer to the value of the bond energy of surface particles $\varepsilon_{p}$ for large monolithic blocks of substances, while in the case of subcolloidal grinding the value of ${\bar{\varepsilon }}_{I}$ will approach the bond energy of the apex particles $\varepsilon_{w}$. The value of this physical magnitude also depends on the mutual ratio of the number of surface particles, edge particles and apex particles. In turn, this dependency is determined by the grain size of the ground substance. The number of apex and edge particles in relation to the number of surface particles increases for smaller grains, thus reducing the average bond energy and, thus, the value of the magnitude α. This means that a representative “average” particle can more easily (lower energy value) be detached from smaller grains than larger ones.

A similar effect of the grain size of the feed can be observed in the case of the volumetric density of the grinding energy. The actual value of the elastic limit stress decreases with a decrease in the average grain size of the feed.

The theoretical value of the elastic limit stress has a constant value. Thus, the decrease in the value of the actual elasticity is the result of an increase in the influence of material defects in the internal structure of the substance, together with a decrease in the grain average size of the feed. The finer the grains, the greater the impact of material defects on the actual value of elastic stresses and the greater the material weakening. The surface interaction energy, determined by the magnitude α, plays a decisive role in the process of grinding fine-grained materials. It has a decisive influence on the magnitude of the energy required for grinding.

## 6. Conclusions

From the general analysis of the modified form of the thermodynamic theory of grinding, it appears that the value of the grinding energy basically depends on two magnitudes—one related to the surface interaction energy (feed granularity) and the other related to the strength properties of the substance and its volume. These two components have different effects on the magnitude of the grinding energy depending on the state of the substance before grinding. The research proved that even slightly different feeds in terms of size distribution are characterized by different susceptibilities to grinding. Thus, averaging the tested material characteristics is a great simplification and leads to an incorrect analysis of the process.

Knowledge of tested material magnitudes is necessary for modeling thermomechanical phenomena in the solid state.

## Data Availability

The data presented in this study are available on request from the corresponding author.

## References

[1] Kulczycka J. (2019). Circular Economy in Politics and Research.

[2] Alhawari O., Awan U., Bhutta M., Khurrum S., Ülkü M.A. (2021). Insights from Circular Economy Literature: A Review of Extant Definitions and Unravelling Paths to Future Research. Sustainability.

[3] Velenturf A.P.M., Purnell P. (2021). Principles for a sustainable circular economy. Sustain. Prod. Consum..

[4] Cieplińska A., Szymanek A. (2019). Waste anthropogenic minerals in the circular economy. J. Phys. Conf. Ser..

[5] Zhukov V.P., Otwinowski H., Belyakov A.N., Wyleciał T., Mizonov V.E. (2015). Boltzmann equation in the modeling of mineral processing. Arch. Min. Sci..

[6] Li Z., Jiang J., Lyu J., Cao J. (2021). Orthotropic Viscoelastic Properties of Chinese Fir Wood Saturated with Water in Frozen and Non-frozen States. For. Prod. J..

[7] Mayer-Laigle C., Blanc N., Rajaonarivony K., Rouau X. (2018). Comminution of Dry Lignocellulosic Biomass, a Review: Part I. From Fundamental Mechanisms to Milling Behaviour. Bioengineering.

[8] Romanovich A.A., Romanovich L.G., Chekhovskoy E.I. (2018). Determination of rational parameters for process of grinding materials pre-crushed by pressure in ball mill. IOP Conf. Ser. Mater. Sci. Eng..

[9] Ciobanu C., Tudor P., Constantin G.-A., Musuroi G. (2020). Determination of granulometrical composition of the clinker by grinding in a ball mill to determine the specific consumption of additional energy. E3S Web Conf..

[10] Kamarova S., Abildinova S., Terziev A., Elemanova A. (2020). The efficiency analysis of the SH-25A ball drum mill when grinding industrial products of fossil fuels. E3S Web Conf..

[11] Yin Z., Peng Y., Zhu Z., Yu Z., Li T. (2017). Impact load behavior between different charge and lifter in a laboratory-scale mill. Materials.

[12] Tukarambai M., Hemanth Varma M.S., Raju C.A.I. (2020). Batch grinding studies by a ball mill for hematite ore. Mater. Today Proc..

[13] Yang J., Shuai Z., Zhou W., Ma S. (2019). Grinding optimization of cassiterite-polymetallic sulfide ore. Minerals.

[14] Yu J., Qin Y., Gao P., Han Y., Li Y. (2021). An innovative approach for determining the grinding media system of ball mill based on grinding kinetics and linear superposition principle. Powder Technol..

[15] Bakker J.D. (2014). Energy use of fine grinding in mineral processing. Metall. Mater. Trans. E.

[16] Santosh T., Rahul K.S., Eswaraiah C., Rao D.S., Venugopal R. (2020). Optimization of stirred mill parameters for fine grinding of PGE bearing chromite ore. Particul. Sci. Technol..

[17] Akkaya B., Toroğlu I., Bilen M. (2020). Studying the effect of different operation parameters on the grinding energy efficiency in laboratory stirred mill. Adv. Powder Technol..

[18] Guo W., Han Y., Gao P., Li Y., Tang Z. (2021). Effect of feed size on residence time and energy consumption in a stirred mill: An attainable region method. Powder Technol..

[19] Duan J., Lu Q., Zhao Z., Wang X., Zhang Y., Wang J., Li B., Xie W., Sun X., Zhu X. (2020). Grinding behaviors of components in heterogeneous breakage of coals of different ash contents in a ball-and-race mill. Minerals.

[20] Yang Y., He Y., Bi X., Grace J.R., Wang H., Fotovat F., Xie W., Wang S. (2020). Effect of moisture on energy-size reduction of lignite coal in Hardgrove mill. Fuel.

[21] Pareek P., Sankhla V.S. (2021). Review on vertical roller mill in cement industry & its performance parameters. Mater. Today Proc..

[22] Avalos S., Kracht W., Ortiz J.M. (2020). Machine learning and deep learning methods in mining operations: A data-driven SAG mill energy consumption prediction application. Mining Metall. Explor..

[23] Pamparana G., Kracht W., Haas J., Ortiz J.M., Nowak W., Palma-Behnke R. (2019). Studying the integration of solar energy into the operation of a semi-autogenous grinding mill. Part I: Framework, model development and effect of solar irradiance forecasting. Miner. Eng..

[24] Pamparana G., Kracht W., Haas J., Ortiz J.M., Nowak W., Palma-Behnke R. (2019). Studying the integration of solar energy into the operation of a semi-autogenous grinding mill. Part II: Effect of ore hardness variability, geometallurgical modeling and demand side management. Miner. Eng..

[25] Behnamfard A., Namaei R.D., Veglio F. (2020). The performance improvement of a full-scale autogenous mill by setting the feed ore properties. J. Clean. Prod..

[26] Orekhova T.N., Sheremet E.O., Kachaev A.E. (2020). Analysis of power parameters of a rotary mill. IOP Conf. Series. Mater. Sci. Eng..

[27] Chițoiu M., Voicu G., Moiceanu G., Paraschiv G., Dinca M., Vladut V., Tudor P. (2018). Energy consumption analysis on energetic plant biomass grinding using hammer mills. UPB Sci. Bull. Ser. D.

[28] Eliseev M.S., Zagoruyko M.G., Rybalkin D.A., Leontyev A.A., Peretyatko A.V. (2018). Determination of speed range of Hammer mill grinder. ARPN J. Eng. Appl. Sci..

[29] Moiceanu G., Paraschiv G., Voicu G., Dinca M., Negoita O., Chițoiu M., Tudor P. (2019). Energy consumption at size reduction of lignocellulose biomass for bioenergy. Sustainability.

[30] Liu Y., Wang J., Barth J.C., Welsch K.R., McIntyre V., Wolcott M.P. (2020). Effects of multi-stage milling method on the energy consumption of comminuting forest residuals. Ind. Crops Prod..

[31] Kruszelnicka W., Kasner R., Bałdowska-Witos P., Flizikowski J., Tomporowski J. (2020). The integrated energy consumption index for energy biomass grinding technology assessment. Energies.

[32] Bulgakov V., Pascuzzi S., Ivanovs S., Kaletnik G., Yanovich V. (2018). Angular oscillation model to predict the performance of a vibratory ball mill for the fine grinding of grain. Biosyst. Eng..

[33] Mayer-Laigle C., Blanc N., Rajaonarivony K., Rouau X. (2018). Comminution of Dry Lignocellulosic Biomass: Part II. Technologies, Improvement of Milling Performances, and Security Issues. Bioengineering.

[34] Tumuluru J.S., Heikkila D.J. (2019). Biomass grinding process optimization using response surface methodology and a hybrid genetic algorithm. Bioengineering.

[35] Ogonowski S., Ogonowski Z., Pawełczyk M. (2018). Multi-Objective and Multi-Rate Control of the Grinding and Classification Circuit with Electromagnetic Mill. Appl. Sci..

[36] Rittinger P.R. (1867). Lehrbuch der Aufbereitungskunde in Ihrer Neuesten Entwicklung und Ausbildung.

[37] Kick F. (1883). Contributions to the knowledge of the mechanics of brittle materials. Dinglers Polytech. J..

[38] Kick F. (1883). The law of proportional resistances and its application to pressure in sand and explosions. Dinglers Polytech. J..

[39] Kick F. (1885). Das Gesetz der Proportionalen Widerstände und Seine Anwendun.

[40] Bond F.C. (1952). The third theory of comminution. AIME Trans..

[41] Bond F.C. (1960). Confirmation of the third theory. AIME Trans..

[42] Bond F.C. (1962). Crushing and Grinding Calculations.

[43] Charles R.J. (1956). High velocity impact in comminution. Min. Eng..

[44] Charles R.J., Bruyn P.L. (1956). Energy transfer by impact. Trans. AIME..

[45] Charles R.J. (1957). Energy-size reduction relationships in comminution. Min. Eng..

[46] Djingheuzian L.E. (1949). A study of present day grinding. Can. Min. Metall. Bull..

[47] Djingheuzian L.E. (1957). A Study of Operating Data from Ball Mills Operating in Quebec, Ontario, Manitoba and British Columbia. Can. Min. Metall. Bull..

[48] Djingheuzian L.E. (1954). The influence of temperature on efficiency of grinding. Can. Min. Metall. Bull..

[49] Guillot R. (1960). Le Problème du Broyage et Son Evolution.

[50] Mielczarek E. (1982). Free Comminution of Brittle Solid Substances.

[51] Mielczarek E. (1984). Particle size distribution of free comminution product. Arch. Min. Sci..

[52] Stamboliadis E.T. (2002). A contribution to the relationship of energy and particle size in the comminution of brittle particulate materials. Miner. Eng..

[53] Jung H., Lee Y.J., Yoon W.B. (2018). Effect of Moisture Content on the Grinding Process and Powder Properties in Food. A Review. Processes.

[54] Hukki R.T. (1961). Proposal for a solomonic settlement between the theories of von Rittinger, Kick and Bond. Trans. AIME.

[55] Rumpf H. (1973). Physical aspects of comminution and new formulation of a law of comminution. Powder Technol..

[56] Duroudier J.P. (2016). Size Reduction of Divided Solids.

[57] Jiang S., Tang C., Li X., Tan Y., Peng R., Yang D., Liu S. (2020). Discrete element modeling of the machining processes of brittle materials: Recent development and future prospective. Int. J. Adv. Manuf. Technol..

[58] Zawada J. (1998). Introduction to the Mechanics of Crushing Processes.

[59] Griffith A.A. (1928). The phenomena of Rupture and Flow in Solid. Phil. Trans. Roy. Soc..

[60] Mielczarek E. (1982). Characteristic solid of the unit energy consumption of grinding. Arch. Mech. Eng..

[61] Górecka-Zbrońska A., Otwinowski H., Zbroński D. (2003). Research on the Single Impact Comminution Mechanism of Quartz Sand in the Jet Mill. Powder Handl. Process..

[62] Urbaniak D. (2002). Determination of the free energy intensity of free grinding of brittle materials. Sci. Work. Wars. Univ. Technol. Conf..

[63] Urbaniak D., Wyleciał T. (2010). Mechanical Activation in Energy Processes. Chem. Process Eng..

